# Knowledge, Attitude and Practices Towards COVID-19 Vaccination Among Students and Staff at the Islamic University in Uganda

**DOI:** 10.24248/eahrj.v7i2.740

**Published:** 2023-11-30

**Authors:** Naziru Rashid, Madinah Nabukeera, Zubair Jauhara Kisambira, Swaibu Zziwa, Mariam Abbasi Ndagire, Faridah Nakayiza

**Affiliations:** a Department of Public Health and Community Medicine, Faculty of Health Sciences, Islamic University in Uganda; b Faculty of Management Studies, Islamic University in Uganda; c Faculty of Medicine, Gulu University, Uganda; d Faculty of Health Sciences, Islamic University, Uganda; e Uganda National Health Laboratory Services

## Abstract

**Background:**

Whereas the burden of Coronavirus Disease of 2019 (COVID-19) has greatly reduced worldwide in terms of morbidity and mortality, its impact is still affecting many countries economically, politically as well as in the healthcare systems. There is therefore a need to ensure that the community is protected and safe against the resurgence of the COVID-19 by embracing the vaccination against the disease. Taking up campaign messages about the COVID-19 vaccine, requires the community to have a certain level of understanding and have a good or positive attitude towards the vaccines, all of which influence good practices.

**Aim of the Study:**

This study therefore, aimed at establishing the knowledge, attitude and practices towards COVID-19 vaccination among students and staff at the Islamic University in Uganda (IUIU).

**Methods:**

The study used a cross-sectional design, and employed quantitative methods to collect data with semi-structured questionnaire. Data were analyzed using the IBM SPSS Statistics for Windows, Version 27.0. (IBM Corp, Armonk, NY, USA).

**Results:**

A total of 397 respondents participated in the study. The mean knowledge score on COVID-19 Vaccination was 57.5%. All respondents had ever heard about COVID-19 vaccine. More than half of the respondents (54.2%) knew the vaccination site for COVID-19 vaccine. Few respondents (38.5%) knew the different brands of COVID-19 Vaccines. Only 34.1% of the respondents agreed that the COVID-19 vaccine was very effective; while 37.8% of the respondent's preferred vaccination as a control measure for COVID-19, and the rest preferred other means including face masks and hand washing.

**Conclusion:**

Health education targeting the safety and efficacy of the vaccine will most likely impact knowledge, attitude and practice of respondents and eventual uptake, especially if messaging comes from trusted sources.

## BACKGROUND

Coronaviruses are a large group of viruses that are rather common throughout the world.^1,2^. Evidence has shown that the virus is transmitted through birds and mammals, with humans being particularly vulnerable to the infection and transmission of the virus.^2,3^ Cases of pneumonia-like illness of unknown cause were observed in Wuhan city in China and reported to the World Health Organization (WHO) which subsequently declared it a public health emergency of international concern and a global pandemic on 30^th^ January 2020 and 11^th^ March 2020 respectively.^4,5^ The disease is characterized by dry cough and shortness of breath with difficulty in breathing and at least 2 of the following; fever, chills, muscle pain, headache, sore throat and loss of taste and smell. The pathogen responsible for the disease being severe acute respiratory syndrome coronavirus-2 (SARS-CoV-2).^4^

Whereas the burden of COVID-19 has greatly reduced worldwide in terms of morbidity and mortality, its impact is still affecting the economic, political and the healthcare systems of many countries striving to achieve sustainable development goal number 3 (SDG-3) targets on good health and wellbeing. Total number of deaths directly attributed to COVID-19 by mid-August of 2023 was 6.9 million with 769 Confirmed cases.^3^ In Africa, 9.5 million people have been confirmed to be COVID-19 positive, with Uganda reporting 171,729 cases and more than 3,632 deaths by August 2023.^6^ This pandemic also affected many essential services including increase in psychiatric cases of anxiety and depression, lowering of life expectancy, as well as affecting the control of HIV,TB and malaria control.^7^

As a result, immunisation coverage dropped for the first time in 10 years, and deaths from TB and malaria increased. Urgent and concerted action is needed to set the world back on a trajectory towards achieving SDG number 3.^8^

Thus, countries and healthcare service providers need to re-plan and re-strategize on how best to re-focus their efforts towards achieving SDG number 3 in the post COVID-19 pandemic period. This also requires that individual institutions and communities prevent the resurgence of the pandemic by adherence to the prevention of COVID-19 control measures especially vaccination. For any vaccination campaign to be effective and achieve herd immunity, a critical mass of the population should be vaccinated;^9^ which depends on the knowledge and perception of the community about the vaccines.^10^ In Uganda, previous studies have found the prevalence of COVID-19 vaccination to be as low as 20.4% among university students and staff.^9^ Several factors including knowledge, attitude and perceptions are responsible for the community's willingness to take up vaccination. Since there is presently no widely recommended effective treatment for COVID-19, vaccination is the biggest hope for the control of the COVID-19 pandemic. Vaccination has been effective in the prevention of infectious diseases thereby reducing associated morbidity and mortality and it is a cornerstone for prevention and control of infectious diseases. However, the success of vaccination against COVID-19 will be largely dependent on the willingness of the general population to accept and receive the vaccines for the subsequent development of herd immunity.^11^

The WHO has recommended the Oxford/AstraZeneca COVID-19 vaccine and other vaccines for use in all adults. It has recommended two doses of the vaccine to be given 8 to 12 weeks apart. Three COVID-19 vaccines are also authorized and approved for use in the United States to prevent COVID-19 including Pfizer-BioNTech or Moderna (COVID-19 mRNA vaccines).^12^ A recent study on COVID-19 vaccine efficacy in Israel revealed that Pfizer/BioNTech's vaccine is about 85% effective after the first dose.^13^

Uganda launched its mass COVID-19 vaccination program in March 2021, but uptake of COVID-19 vaccination is still low.^9,14^ According to WHO, Uganda has administered about 26 million doses of COVID-19 vaccines by 16^th^ August 2023.^6^ Much as vaccines are essential in battling against COVID-19, it is of paramount importance to assess knowledge, attitude and perception towards COVID-19 vaccine before they are introduced to the community.^14^ Therefore, the present study intended to establish the knowledge, attitude and perception towards COVID-19 vaccination among students and staff at IUIU in order to establish evidence-based interventions to increase uptake of COVID-19 vaccine. The University is an international university attracting staff and students from different parts of Africa and different regions within Uganda. This not only puts the University at a risk of importing cases but also it is a good representation of the general community.

## METHODOLOGY

### Study Setting

The study site was IUIU, which is the second oldest and the first Private University in Uganda, East Africa. It has 4 Campuses; the main campus in Mbale District in the eastern region of Uganda, Kampala campus at Kibuli in Kampala district, Females' campus in Wakiso district both in central Uganda and Arua campus in the North Western part of Uganda. As of 2020/2021 Academic Year the University had an enrollment of 9,280 students in all its four Campuses and 1,258 members of staff. The university has 7 faculties including faculty of Health sciences, law, education, science, management studies, and faculty of Arts and Social Sciences. The University hosts students from 27 countries of Africa. At the time of data collection, the students were not physically at campus due to closure of Academic Institutions by the government of Uganda as measure of controlling COVID-19. As a result, data was collected through an online survey.

### Study Design

A cross-sectional study design was conducted between July and October 2021. Quantitative methods were used to collect data from study participants.

### Study Population

The study population included students and staff currently enrolled at all the four campuses of IUIU. These students and staff were from all the faculties and not necessarily health sciences only.

### Inclusion and Exclusion Criteria

IUIU Students and staff were electronically requested to consent to participate in the study; all those who consented were automatically included in the study.

### Sample Size Determination

The following simple formula by Daniel, 1999 was used to determine the sample size;^15^



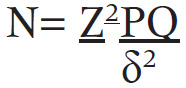



Where:

N is our sample size that we are looking for.

Z is the standard normal deviation at 95% confidence equal to 1.96

P is estimated prevalence of vaccination uptake, for our case we used 57.8% the prevalence of COVID-19 vaccination uptake among healthcare workers in Wakiso district.(16)

Therefore, our P = 0.578, and Q is equal to 1-P = 1-0.578= 0.422

δ is our maximum acceptable error and was taken as 0.05 Therefore, our sample size is [(1.96×1.96) × 0.578×0.422] / [0.05×0.05] = 375 participants. Plus 10% Non-compliance, gives us a total sample size of 412.

### Sampling Procedure

Simple random sampling was used to select all students and staff of IUIU who could be accessed through emails, WhatsApp and ERP electronic system using computer generated random numbers. The list of students and staff was obtained from university records office.

### Study Variables Variables

**Dependent Variable:** Knowledge, attitude and practices towards COVID-19 vaccine.

**Independent Variables:** Age category, sex, education status, source of income, religion, marital status, nationality risk perception, attitude on COVID-9 vaccines including fear of side effects and vaccine efficacy the vaccines and barriers to accessing the vaccines.

### Data Collection Procedure and Tool

A structured questionnaire was pre tested by administering it to to10 students and staff of IUIU and issues raised were considered and corrected.

The questionnaire and the link to the questionnaire was then shared to students and staff of IUIU through the University ERP-system (the electronic management system for IUIU), official and personal emails, and the different WhatsApp groups.

### Quality Assurance and Control Training of Research Assistants

There was no need of research assistants since data was collected online

### Field Editing of data

Data was constantly cleaned during the data collection period.

### Data Management and analysis

Data cleaning and validation was done and data entered into excel sheets and analysis was performed using the IBM SPSS Statistics for Windows, Version 27.0. Armonk, NY: IBM Corp released in 2020. All socio-demographic study variables were presented in percentage, mean, and standard deviation.

### Data Analysis

Data collected from questionnaires were entered in Microsoft excel and exported to SPSS. Proportions of the study variables such as age and sex were computed and presented in frequency tables. Using Blooms score for categorization of knowledge and attitudes, questions to determine the knowledge of respondents towards COVID-19 were scored and categorized as low knowledge (1–10) and high knowledge (11–18). Attitudes of respondents towards COVID-19 was measured using a four-point Likert scale with responses ranging from 1 (strongly disagree) to 4 (strongly agree) and responses were further grouped into a dichotomous scale “agree” and “disagree” Those who had selected strongly agree and agree were categorized as “agree” and those who had selected disagree and strongly disagree were categorized as “disagreeing”.

Questions about practices of respondents towards COVID-19 were scored and categorized as improper practices (1–6) and proper practices (7–10).

### Ethical Consideration.

Permission to conduct the study with in the university was granted by the university secretary through the director research, publication and innovation office (RPI). While ethical approval was obtained from the IUIU Research Coordination Committee (RCC) under Ref. *Number RCC/FHS/21/006.* All participants were made to sign an informed consent (electronically) after fully understanding the details of the study. Participants identification was not used anywhere in our research tools thus ensuring anonymity of our participants. There were no direct benefits or potential risks to the participants related to this study.

### COVID-19 Risk Management

Data Collection was done online through Google forms as a measure of controlling direct contact with the participants to minimize the possibility of spreading COVID-19.

## RESULTS

### Demographics

The study looked at the demographic characteristics of the respondents who participated in the study (Table 1). The results indicate that most of the respondents (49.1%) were from the Main campus (Mbale), followed by 39.8% of the respondents from Kampala campus, and the least (4.5%) respondents were from the Females' campus. Majority of the respondents (56.7%) were males as compared to 42.8% of their female counterparts; while 0.5% of the respondents preferred not to disclose. A bigger proportion (58.7%) of the study participants were aged 18–27 years, followed by 25.9% of the participants aged 26–37 years, and the least proportion (1.3%) of the participants were aged above 57 years. Majority of the participants (69.5%) were Muslims, followed by Catholics (11.6%), Anglican Protestants (10.8%), and Seventh Day Adventists who were the least at 0.8%. The results also indicate that the majority (78.3%) of the study participants were students; with the staff constituting 21.7%, of which the teaching staff were the most (54.7%), and the rest (45.3%) being non-teaching staff.

**TABLE 1:** Demographic Characteristics of Respondents

Category	Frequency (N=397)	Percentage (%)
Campus		
Arua Campus	26	6.5
Females' Campus	18	4.5
Kampala Campus	158	39.8
Main Campus	195	49.1
Total	397	100.0
Gender		
Female	170	42.8
Male	225	56.7
Prefer not to say	2	0.5
Total	397	100.0
Age Category		
18–27 years	233	58.7
28–37 years	103	25.9
38–47 years	41	10.3
48–57 years	15	3.8
Above 57 years	5	1.3
Total	397	100.0
Religion		
Born again (Pentecostal)	25	6.3
Catholic	46	11.6
Muslim	276	69.5
Protestant (Anglican)	43	10.8
Seventh day Adventist	3	0.8
Others	4	1.0
Total	397	100.0
Marital Status		
Divorced	4	1.0
Married	126	31.7
Single	267	67.3
Total	397	100.0
Responsibility held		
Staff	86	21.7
Student	311	78.3
Total	397	100.0
Type of Staff		
Non-Teaching Staff	39	45.3
Teaching Staff	47	54.7
Total	86	100.0

Source: Field data (2021)

### Knowledge and attitude of respondents towards COVID-19 vaccine

The study assessed the respondents' knowledge and attitude towards COVID-19 vaccine (Table 2). When respondents were asked if they had ever heard about COVID-19 vaccine, all (100.0%) respondents had stated that they had ever heard about COVID-19 vaccine. About the part or place where the COVID-19 vaccine is administered, majority of the respondents (54.2%) said on the left upper shoulder, 20.7% on the right upper shoulder, 0.3% on the buttocks, and 24.4% did not know.

**TABLE 2:** Knowledge and attitude on Covid-19 vaccine (N=397)

Knowledge and attitude	Response	Frequency	Percentage (%)
Have you ever heard about Covid-19 vaccine?	Yes	397	100.0
	No	0	0.0
Where is Covid-19 vaccine administered?	Left upper shoulder	215	54.2
	Right upper shoulder	82	20.7
	Right thigh	1	0.3
	Left thigh	1	0.3
	On the buttocks	1	0.3
	I don't know	97	24.4
Preference on vaccine brand. I would wish to take,	Astra Zeneca	88	22.2
	Moderna	11	2.8
	Pfizer-BioNtech	56	14.1
	Johnson & Johnson's Janssen	51	12.8
	I can get any available brand	38	9.6
	I don't know any of the brands	153	38.5
How many doses of Covid-19 vaccine are you expected to receive?	1 dose	10	2.5
	2 doses	226	56.9
	3 doses	54	13.6
	I don't know	107	27.0
What is the time interval between the doses of Covid-19 vaccine?	3 weeks	21	5.3
	4 weeks	44	11.1
	8 weeks	63	15.9
	12 weeks	43	10.8
	8 to 12 weeks	43	10.8
	I don't know	183	46.1
I believe the Covid-19 vaccine is a very effective vaccine	Strongly Agree	53	13.4
	Agree	82	20.7
	Neutral	166	41.8
	Disagree	56	14.1
	Strongly Disagree	30	7.6
Covid-19 vaccine contains harmful substance which can be harmful to our body	Strongly Agree	49	12.3
	Agree	60	15.1
	Neutral	190	47.9
	Disagree	74	18.6
	Strongly Disagree	24	6.0
I believe the Covid-19 vaccine is a very effective vaccine	Strongly Agree	53	13.4
	Agree	82	20.7
	Neutral	166	41.8
	Disagree	56	14.1
	Strongly Disagree	30	7.6
Covid-19 vaccine contains harmful substance which can be harmful to our body	Strongly Agree	49	12.3
	Agree	60	15.1
	Neutral	190	47.9
	Disagree	74	18.6
	Strongly Disagree	24	6.0

Source: Field data (2021)

When respondents were asked about preference on vaccine brand, most of the respondents (38.5%) did not know any of the brands, 22.2% of the respondents preferred Astra Zeneca, followed by 14.1% who preferred Pfizer-BioNTech, then 12.8% preferred Janssen's Johnson & Johnson, and only few (2.8%) preferred Moderna.

The study findings also show that majority of the respondents (56.9%) expected to receive 2 doses of COVID-19 vaccine, 27% did not know, and a few (2.5%) of the respondents expected to receive one dose of the vaccine. The results also indicate that most of the respondents (46.1%) did not know the time interval between the doses of COVID-19 vaccine, 15.9% said 8 weeks, 11.1% four weeks, and the least (5.3%) said three weeks. On the issue of effectiveness of COVID-19 vaccine, 34.1% of the respondents agreed that the COVID-19 vaccine is a very effective vaccine, but 21.7% disagreed. However, most of the respondents (41.8%) were not sure about the effectiveness of the COVID-19 vaccine. Similarly, the results indicate that 27.4% of the respondents agreed that COVID-19 vaccine contains harmful substances which can be harmful to our body, 24.6% of the respondents disagreed, and most of the respondents (47.9%) were not sure.

### Practices of respondents towards COVID-19

The study also assessed the different practices on COVID-19 prevention at IUIU and the results are presented in Table 3. Most of the respondents (37.8%) preferred using a face mask as a control measure of COVID-19, followed by 37.0% of the respondents who preferred vaccination, and then a few respondents (8.6%) preferred frequent hand washing as a control measure of COVID-19.

**TABLE 3:** Practices of Respondents Towards Covid-19 (N=397)

Statements	Response	Frequency	Percentage (%)
Preferred control measures of Covid-19	Frequent hand washing	34	8.6
	Keeping social distance	59	14.9
	Using face mask	150	37.8
	Vaccination	147	37
	I don't know	7	1.8
I frequently put on a face mask as a way of controlling Covid-19	Agree	350	92.1
	Disagree	30	7.9
I frequently avoid social gatherings like congregational prayers, funeral, and market places	Strongly Agree	177	45.4
	Agree	157	40.3
	Undecided	28	7.2
	Disagree	23	5.8
	Strongly Disagree	5	1.3
I frequently wash my hands whenever I enter campus, faculty or department	Agree	359	92.5
	Disagree	29	7.5

Source: Field data (2021)

When respondents were asked whether they frequently put on a face mask as a way of controlling COVID-19, majority of the respondents (92.1%) supported the argument while few (7.9%) of the respondents declined. This implies that respondents frequently wore face masks to control COVID-19. Additionally, the findings indicate that a larger proportion (85.7%) of the respondents agreed that they frequently avoided social gatherings like congregational prayers, funerals, and market places; while only a few (7.1%) of the respondents disagreed. Furthermore, the study findings indicate that majority of the students and staff frequently washed their hands whenever they entered campus, faculty or department since a bigger proportion (92.5) of the respondents supported the argument while only a few (7.5%) of the respondents declined. Generally, a number of practices and measures were undertaken by the students and staff towards the prevention of COVID-19.

## DISCUSSION

### Knowledge and Attitude of the Respondents Towards COVID-19 Vaccination

Participants exhibited some level of knowledge on COVID-19 vaccination with some gaps. This may imply that even the educated did not have clear knowledge on the COVID-19 vaccine since they relied on social media which provided negative information. There is still gaps in knowledge about the COVID-19 vaccine among the educated in Uganda in spite of the country wide mobilization campaigns hence affecting the attitude and practices towards prevention and spread of Covid-19. This group of participants were at a high risk since they had moderate information on the Covid-19 vaccine. This in the end would affect Covid-19 vaccine acceptability rates. It may also imply that government has not done enough in line with sensitization, education, transparency, inclusive participation and communication about Covid-19 vaccine.

All respondents demonstrated the fact they had heard about the vaccines before. This suggests that there was considerable efforts made to communicate information about COVID-19 vaccination for public awareness Though the government still needs to further sensitize the public to understand the need for COVID-19 vaccination and the part where the vaccines should be administered since in the university a small percentage didn't know the correct site for the vaccine to be administered. The study found that Astra Zeneca and Pfizer-BioNTech were the most preferred vaccine brands by participants. Thus the government needs to invest more in procuring the above brands to enable adherence of the community to COVID-19 vaccination.^21^

Majority of the participants were not aware of the time intervals between the first and the second doses of COVID-19 vaccines. If not addressed this can affect adherence and compliance to getting the second dose of vaccination. Public sensitization still needed to enable adherence to the second dose.^22^ However, most of the respondents (41.8%) were not sure about the effectiveness of the COVID-19 vaccine and a good number thought that the vaccines contained toxic and harmful substances This implies that there is a lot of misperceptions about the effectiveness and harmfulness of the COVID-19 vaccine. These misconceptions increase the chances of staff and university students to distance from themselves from vaccination.^23,24^ It is thus important for government to disseminate any necessary information through ministry of health on the effectiveness of the vaccines; this will increase chances of people getting vaccinated.^23^

### Practices of respondents towards COVID-19 prevention

Most of the respondents preferred using a face mask and handwashing as control measures of COVID-19, compared to vaccination. This finding was similar to the earlier findings of Naziru who found that majority of the people in the community preferred use of facemasks as a control measure of COVID-19.^4^ This shows that students and staff of IUIU were probably complying with the implementation of Standard Operating Procedures as recommended by government, COVID-19 taskforce, ministry of health, Ministry of Education, Inter-University Council of East Africa and National Council for Higher Education.^25^ Generally, a number of practices and measures were undertaken by the students and staff towards the prevention of COVID-19. The study findings are similar with those of Hossain and others.^17^

## CONCLUSION

The participants of this study had moderate knowledge regarding COVID-19 vaccination. The mean knowledge score was 57.5%. All the respondents (100.0%) had ever heard about COVID-19 vaccine. Whereas most of the respondents preferred Astra Zeneca, and Pfizer as their choice of vaccine brand a good number still did not know the different brands. Thus, government needs to invest more in those brands of choice and also increase sensitization of the community on safety and effectiveness of the vaccines.

## RECOMMENDATIONS

Basing on the study findings, we recommend the following;

More attention needs to be focused on health education and promotion to change the misconceptions about the COVID-19 vaccine.

Health experts should ensure that required information is comprehensively explained to the participants and that the important aspects of study participation are emphasised.

The University need to focus attention on health education campaigns targeted at both students and staff to improve the knowledge and uptake of the vaccine.

Continuous health education of students and staff through journal clubs, mass media particularly on the effectiveness of COVID-19 vaccine.

There is a need of campaigns to improve preventive practices such as physical distancing, handwashing and respiratory etiquette.

### Study Limitations

Online data collection method may have excluded students and staff who could not access internet services from participating in the study.
